# Geographic Disparities in Gynecologic Oncology Clinical Trial Availability in the US

**DOI:** 10.1001/jamanetworkopen.2024.47635

**Published:** 2024-11-26

**Authors:** Mary Regina Boland, Elizabeth Tubridy, Sebastian Spataro Solorzano, Fiona Simpkins, Anna Jo Bodurtha Smith, Emily M. Ko

**Affiliations:** 1 Department of Data Science in Mathematics, Herbert W. Boyer School of Natural Sciences, Mathematics, and Computing, Saint Vincent College, Latrobe, Pennsylvania; 2 Department of Marketing, Analytics and Global Commerce, Saint Vincent College, Latrobe, Pennsylvania; 3 Perelman School of Medicine, University of Pennsylvania, Philadelphia; 4 Division of Gynecologic Oncology, Department of Obstetrics and Gynecology, University of Pennsylvania, Philadelphia; 5 Department of Biosciences, Rice University, Houston, Texas; 6 Leonard Davis Institute of Health Economics, University of Pennsylvania, Philadelphia; 7 Population Aging Research Center, University of Pennsylvania, Philadelphia; 8 Penn Center for Cancer Care Innovation, University of Pennsylvania, Philadelphia

## Abstract

**Importance:**

Disparities in minoritized racial and ethnic populations’ participation in gynecologic cancer clinical trials are well documented despite the high rates of endometrial cancer in these populations. Geographic proximity to trials is a critical component to ensure equitable trial access.

**Objective:**

To characterize the geographic distribution of gynecological cancer trials across the US and identify disparities.

**Design, Setting, and Participants:**

This study is a cross-sectional analysis of trials first posted on ClinicalTrials.gov from January 1, 2013, through January 10, 2024. This study involved a state-level analysis of clinical trials located in the US. Enrollment criteria of clinical trials for ovarian, uterine, cervical, endometrial, vaginal and/or vulvar, and other gynecological cancers were reviewed to exclude nongynecological cancers (1643 trials) or noninvasive gynecological conditions (224 trials).

**Exposure:**

The number of gynecological trials per 100 000 persons in each state.

**Main Outcomes and Measures:**

A state-level analysis was performed to determine whether gynecologic cancer clinical trial availability in the US is associated with other state-level characteristics to identify areas of increased need. Census data, state-level total population size, percentage of non-Hispanic White persons, and the Federal Emergency Management Agency expected annual loss per state as a measure of social vulnerability were aggregated. The association between these variables and the number of gynecological trials per 100 000 persons was measured using Spearman rank correlation.

**Results:**

Of the 1561 invasive gynecological cancer trials that met the inclusion criteria, most cancer trials were ovarian (911 trials [58.4%]), followed by cervical (438 trials [28.1%]), and endometrial (385 trials [24.7%]). Predominantly minoritized population–serving states (ie, those with <50% non-Hispanic White persons) had fewer than 4 trials per 100 000 persons, but this was not significant nationally (ρ = 0.20; 95% CI, −0.08 to 0.45; *P* = .16). States with higher Federal Emergency Management Agency expected annual loss had lower numbers of gynecological trials per 100 000 persons, which was significant nationally (ρ = −0.53; 95% CI, −0.70 to −0.29; *P* < .001).

**Conclusions and Relevance:**

In this cross-sectional study of female gynecological cancer trials by state, states with particularly high economic vulnerability and minoritized populations had low clinical trial availability. Further efforts are needed to address disparities identified in this study to ensure equitable trial access.

## Key Points

**Question:**

Where are gynecological cancer trials found in the US, and do disparities in location exist?

**Findings:**

This cross-sectional study of 1561 gynecological cancer trials from ClinicalTrials.gov found that states with higher Federal Emergency Management Agency expected annual loss had lower numbers of gynecological trials per 100 000 persons and that predominantly minoritized population–serving states (ie, those with <50% non-Hispanic White persons) had fewer than 4 trials per 100 000 persons.

**Meaning:**

Efforts are needed to address the disparities identified in this study, especially the low clinical trial availability in states with particularly high economic vulnerability and minoritized populations.

## Teaser

This cross-sectional study examines the geographic distribution of gynecological cancer trials across the US and identifies disparities in access to such trials.

## Introduction

Each year (as of 2024) in the US, over 110 000 new cases of gynecological cancer will be diagnosed, including malignant neoplasms in the cervix, endometrium, uterus, ovary, fallopian tubes, peritoneum, vagina, ovary, and vulva.^1^ Gynecological cancer also results in an estimated more than 30 000 deaths.^1^ Unfortunately, gynecological cancer disproportionately impacts patients from marginalized, minoritized racial and ethnic communities. Cervical, endometrial, and ovarian cancer mortality are all considerably higher for Black persons compared with persons from other racial and ethnic groups.^2^ Increasing awareness over these disparities has led to a new initiative from the Biden administration focused on women’s health and specifically on funding for women’s cancers.^3^ Access to treatment for gynecologic cancer is disproportionate across racial and ethnic groups, with Black and Hispanic or Latinx patients being the least likely to undergo care, further exacerbating disparities.^4^

There is great interest within medical, academic, and governmental organizations to improve access for oncologic clinical trials to improve health care outcomes in the US.^5^ The main reason for this stems from the potential of clinical trials to bridge—or at least alleviate—gaps of disparity when it comes to cancer treatment and outcomes. Regarding cases specific to gynecologic oncology, previous studies^6,7^ have shown that when White persons and Black persons receive the same treatment within the context of an ovarian cancer clinical trial, they exhibit similar and improved outcomes.

Economic factors are also important to consider because trial enrollees who are wealthier may have access to other resources that may mitigate the health burden of cancer. Furthermore, socioeconomic disparities can sometimes appear as racial disparities and vice versa. Studies have found that racial disparities between White and Black persons were affected by socioeconomic status using area deprivation indices.^8^

Enrolling minoritized populations is challenging in the US because of its history regarding race, ethnicity, and health care, which includes a dark past.^9,10^ Some studies suggest that enrollment for gynecological cancer clinical trials is lower for minoritized groups of patients, which directly affects the generalizability of novel care.^11,12^ Although some studies focus on increasing trial enrollment of persons from minoritized groups,^13^ including matching patients with practitioners and nurses by race and ethnicity,^14^ this can be challenging because trials are not always available in the areas where minoritized groups live. These geographic disparities form the subject of our current study.

Current literature suggests that there are geographic disparities when it comes to clinical trial access.^15^ These geographic disparities could occur for a variety of reasons that remain largely unknown and understudied. Clinical trials are very expensive to conduct, and it is possible that companies choose locations where they expect higher enrollment rates, thereby indirectly leading to reduced trials in minoritized population areas. However, to our knowledge, there have been no studies that analyze this association of potential geographic disparities for gynecologic oncology clinical trials on a national basis across the US. In this article, we aim to explore the role of patient geographic region and social vulnerability on racial disparities with enrollment into gynecologic oncology clinical trials in the US.

## Methods

### Study Design

**Figure 1.**
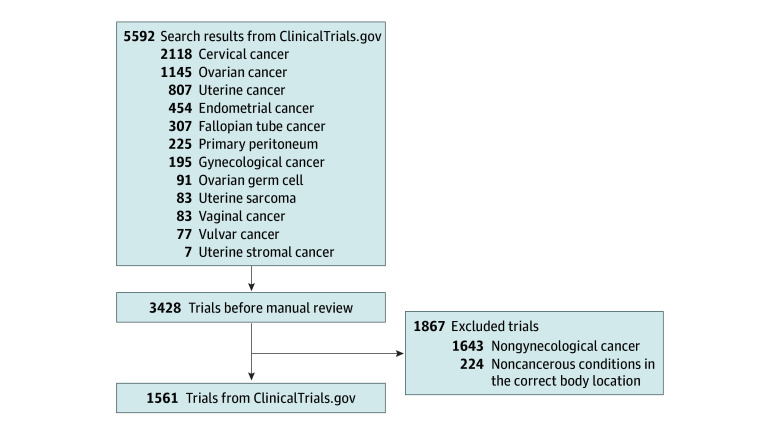
Identification of Gynecological Cancer Clinical Trials Throughout the US from ClinicalTrials.gov Flow diagram shows the inclusion and exclusion of clinical trials identified from ClinicalTrials.gov for geographic mapping. Note that noncancerous conditions such as dysplasia and preinvasive studies were excluded (224 trials).

This cross-sectional study was reviewed by the institutional review board at the University of Pennsylvania and was determined to be exempt from review because of the use of deidentified publicly available data, in accordance with 45 CFR §46. This study follows the Strengthening the Reporting of Observational Studies in Epidemiology (STROBE) reporting guidelines for cross-sectional studies. We performed a cross-sectional analysis of National Institutes of Health ClinicalTrials.gov by retrieving all trials first posted from January 1, 2013, through January 10, 2024. We searched for 12 cancer types: ovarian, uterine, cervical, endometrial, gynecological, uterine stromal, ovarian germ cell, uterine sarcoma, fallopian tube, primary peritoneal, vaginal, and vulvar. Details on the search criteria are shown in the eAppendix in Supplement 1. Next, we grouped these trials into 6 larger gynecological cancer categories (Figure 1): ovarian, uterine, cervical, endometrial, vaginal and/or vulvar, and gynecological cancer.

This resulted in an initial set of 3428 unique clinical trials. We annotated each trial on the basis of the cancers investigated. Therefore, a trial could belong to multiple categories or disease sites (eg, uterine and ovarian cancer) and was counted in each applicable disease site. All 3428 clinical trials were reviewed to ensure correct annotation. We manually reviewed enrollment criteria to exclude nongynecological cancers (1643 trials [47.9%]) or noninvasive gynecological conditions (224 trials [6.5%]). We excluded trials that did not include invasive-gynecologic cancer conditions, such as uterine fibroids, lower genital tract dysplasia, or hyperplasia.

We added some additional annotation labels to our dataset. We labeled trials as pan-cancer if they investigated nongynecologic cancers such as breast, prostate, and skin, while also investigating a gynecologic cancer (ie, we already excluded trials that were only studying nongynecological cancers). Trials were also annotated as genetic if they were exploring a genetic mechanism (eg, human epidermal growth factor receptor 2 status). We identified 119 genetic trials, 76 of which were drug trials, 14 were biological trials, 3 were diagnostic trials, 2 were behavioral trials, 2 were dietary supplement trials, 2 were procedure trials, and 18 were labeled as other interventions.

### Statistical Analysis

We extracted the site location addresses from our final dataset of clinical trials using the location information provided by ClinicalTrials.gov. We then geocoded these addresses to the precise latitude and longitude coordinates. We then aggregated trials to the state level to compute the number of gynecological trials per state. We excluded all international addresses and restricted our analysis to the US, including the 50 states, District of Columbia, and Puerto Rico. For the purposes of this study, we refer to state as any of the 50 states plus either District of Columbia or Puerto Rico.

We aggregated data at the state level and used state-level total population size as of April 1, 2020,^16^ percentage non-Hispanic White persons in 2020,^17,18^ and the Federal Emergency Management Agency (FEMA) expected annual loss (EAL) per state as a measure of social vulnerability.^19^ We used the state-level percentage of non-Hispanic White persons as a way to detect areas where the number of minoritized persons in a state is higher than other regions in the US. Some racial and ethnic groups are mainly found in certain states within the US. For example, Native Hawaiians are found more frequently in Hawaii. By using the percentage of non-Hispanic White persons, all minoritized persons are grouped together. This enabled us to identify disparities related to being in the minoritized racial group. To normalize the number of gynecological trials per state by the population size of that state, we divided the number of gynecological cancer trials by the total population size per state. Note that this total population was across all sex and gender groups. This provided us with the number of gynecological cancer trials per 100 000 persons in each state. We also extracted the age-adjusted new cancer rates for cervical, uterine, and ovarian cancer among female non-Hispanic White persons for 2020 from the Centers for Disease Control and Prevention. We selected White individuals because this was the largest racial and ethnic group with information available.

We measured the association between these demographic variables and the number of gynecological trials per 100 000 persons using Spearman rank correlation. We used Spearman correlation because the number of gynecological trials per 100 000 persons was determined to not follow a normal distribution using the Shapiro test (*P* < .05 cutoff for normality). Therefore, Spearman ρ is used, and 95% CIs for the Spearman ρ were calculated using the DescTools library’s SpearmanRho function. We used the R statistical software version 4.3.1 (R Project for Statistical Computing) for our analyses.

## Results

### Characteristics of US Gynecological Cancer Clinical Trials

A total of 1561 clinical trials were identified in the US that were focused on investigating a gynecological cancer included in our study (Figure 1 and Table). The majority of the 1561 clinical trials identified were investigating ovarian cancer (eFigure 1 in Supplement 1). The most common trials were ovarian (911 trials [58.4%]) and cervical (438 trials [28.1%]), followed by endometrial (385 trials [24.7%]), uterine (158 trials [10.1%]), and vulva-vaginal (78 trials [5.0%]). We also found that 119 trials (7.6%) were explicitly studying a genetic factor. Some trials were not specific to gynecological cancers, but were very broad and included a lot of other cancers as eligibility criteria; we found that 658 trials (42.2%) fit these criteria of being broad and inclusive of many cancer types. This demonstrates that a large proportion of trials were very broad in terms of inclusion criteria, which is indicative of a type of trial called a basket trial. Basket trials test how a drug performs across a wide variety of cancer types that all share a common genetic biomarker (which the drug is designed to target in some way). This could indicate that trials were investigating a particular genetic cause affecting multiple body locations. The rarest cancer types investigated were vulva-vaginal (78 trials [5.0%]). Gynecological cancer not-specified trials (140 trials [9.0%]) refers to trials that used vague language in their eligibility criteria that was not specific to a particular type of gynecological cancer. This was often the case for surveys and other trials that were not investigating a particular pharmacological drug. The number of trials per year is provided in eFigure 2 in Supplement 1.

**Table.** Attributes of Gynecological Cancer Trials Obtained From ClinicalTrials.gov, January 1, 2013, to January 10, 2024

Attribute	Trials, No. (%) (N = 1561)
Geographic region^a^	
Northeast	751 (48.1)
Midwest	644 (41.3)
South	955 (61.2)
West	686 (44.0)
Disease type^a^	
Ovarian, fallopian tube, or primary peritoneal cancer	911 (58.4)
Cervix	438 (28.1)
Endometrium	385 (24.7)
Uterus	158 (10.1)
Vaginal and/or vulvar	78 (5.0)
Gynecological cancer not otherwise specified	140 (9.0)
Pan-cancer^b^	658 (42.2)
Genetic trials	119 (7.6)
Interventions^c^	
Behavioral	155 (9.9)
Biological	173 (11.1)
Combination product	8 (0.5)
Device	51 (3.3)
Diagnostic test	31 (2.1)
Dietary supplement	8 (0.5)
Drug	760 (48.7)
Genetic	7 (0.5)
Not available	1 (0.1)
Other	166 (10.6)
Procedure	110 (7.1)
Radiation	27 (1.7)
Study type^c^	
Interventional	1382 (88.5)
Observational	175 (11.2)
Expanded access	4 (0.3)
Patient target enrollment^c^	
0	33 (2.1)
1-10	140 (9.0)
11-50	528 (33.8)
51-100	265 (17.0)
101-500	425 (27.2)
501-1000	88 (5.6)
1001-5000	52 (3.3)
5001-10 000	10 (0.6)
10 001-50 000	13 (0.8)
>50 000	3 (0.2)

^a^ Trials in this category may belong to other categories in this table section. Each trial can belong to multiple categories because, for example, a trial can recruit patients from across multiple regions within the US.

^b^ Pan-cancer is defined as including at least 1 cancer that was not gynecological cancer so this could include related cancers such as breast and prostate cancer or distant cancers such as melanoma.

^c^ Trials in this category are unique. One trial belongs to each grouping because, for example, the patient enrollment size belongs to exactly 1 category.

### Identifying Patterns and Geographic Analysis of US Gynecological Cancer Clinical Trials

We geocoded these trials to their specific locations understanding that a particular clinical trial could recruit in multiple locations. Therefore, there was not a one-to-one mapping between a clinical trial and a specific location because one trial could recruit in multiple locations. We found that Texas had the highest number of trials (501 trials) followed by California (454 trials) and New York (427 trials). However, this is not surprising given the large population sizes in those states. Therefore, we calculated the number of trials per 100 000 persons in a given state to adjust for each state’s population size. The state with the highest population-adjusted number of trials was South Dakota (8.60 trials per 100 000 persons), followed by Rhode Island (8.40 trials per 100 000 persons). The lowest numbers were in California (1.10 trials per 100 000 persons), Mississippi (0.98 trials per 100 000 persons), and Puerto Rico (0.47 trials per 100 000 persons).

#### Correlation Between Gynecological Cancer Trial Rates and Age-Adjusted Rates of Key Gynecological Cancers

**Figure 2.**
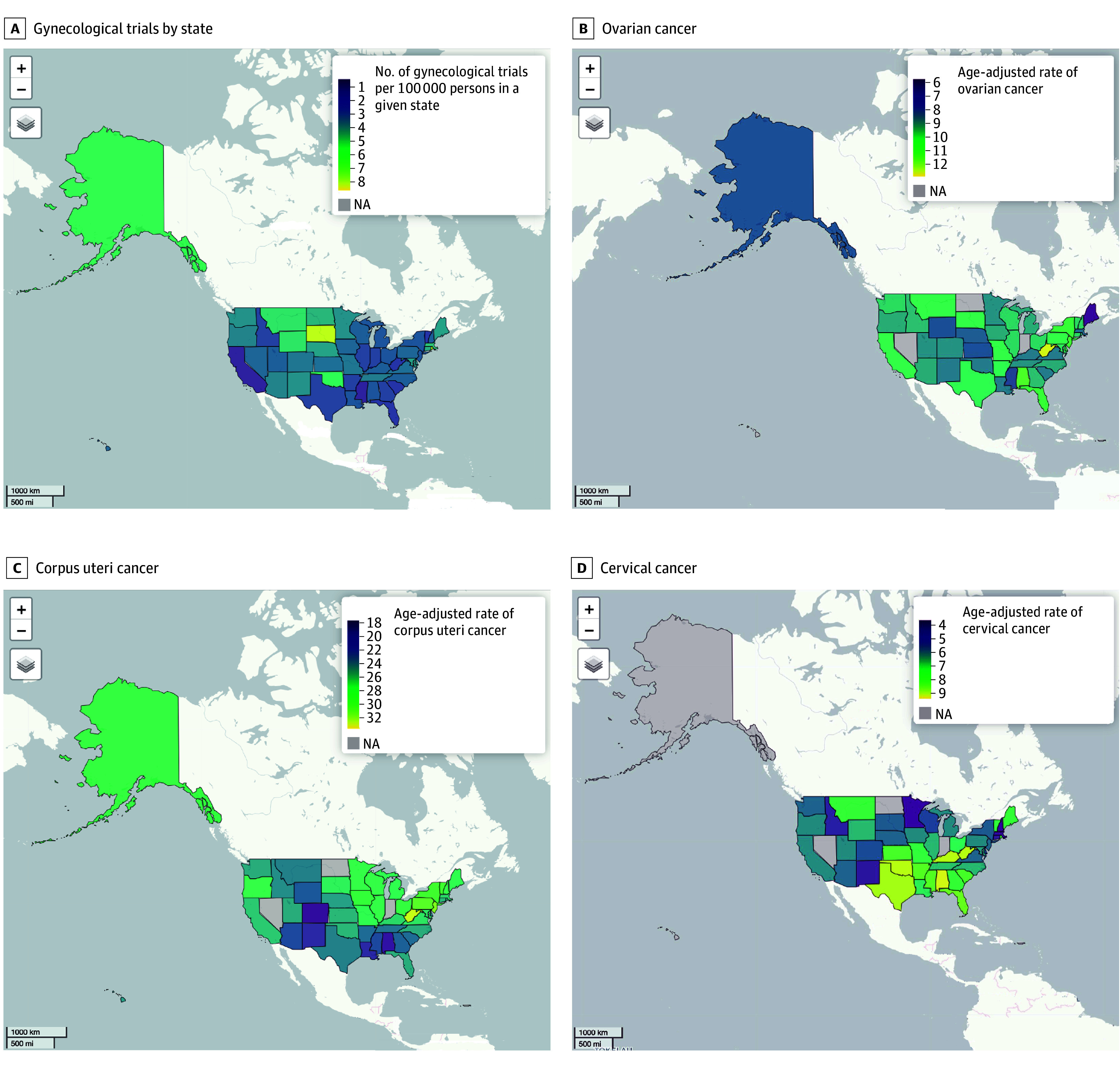
US Maps of Gynecological Cancer Trials Per 100 000 Persons and Age-Adjusted Cancer Rates Maps show the number of gynecological trials per 100 000 persons in a given state (A) and the age-adjusted rates of ovarian cancer (B), corpus uteri cancer (C), and cervical cancer (D) among non-Hispanic White female individuals across the US. NA indicates not applicable. Maps were created in R using OpenStreetMap.

One finding that stands out from Figure 2 is how there appears to be little association between the number of gynecological trials per 100 000 individuals in a given state and the age-adjusted rates of gynecological cancers among female non-Hispanic White persons in those respective states. To test this further, we measured the correlation between the number of gynecological trials per 100 000 individuals in a given state and the age-adjusted rates of gynecological cancers in those respective states and found a significant nonlinear correlation with the number of gynecological trials per 100 000 individuals and age-adjusted cervical cancer (ρ = −0.32; 95% CI, −0.56 to −0.02; *P* = .03) and ovarian cancer (ρ = −0.32, 95% CI, −0.56 to −0.03; *P* = .03), whereas the correlation with corpus uteri cancer (ρ = 0.002; 95% CI, −0.28 to 0.29; *P* = .99) was not statistically significant. Those results are shown in eFigure 3 in Supplement 1.

#### Association With Disparities: Race and Social Vulnerability

**Figure 3.**
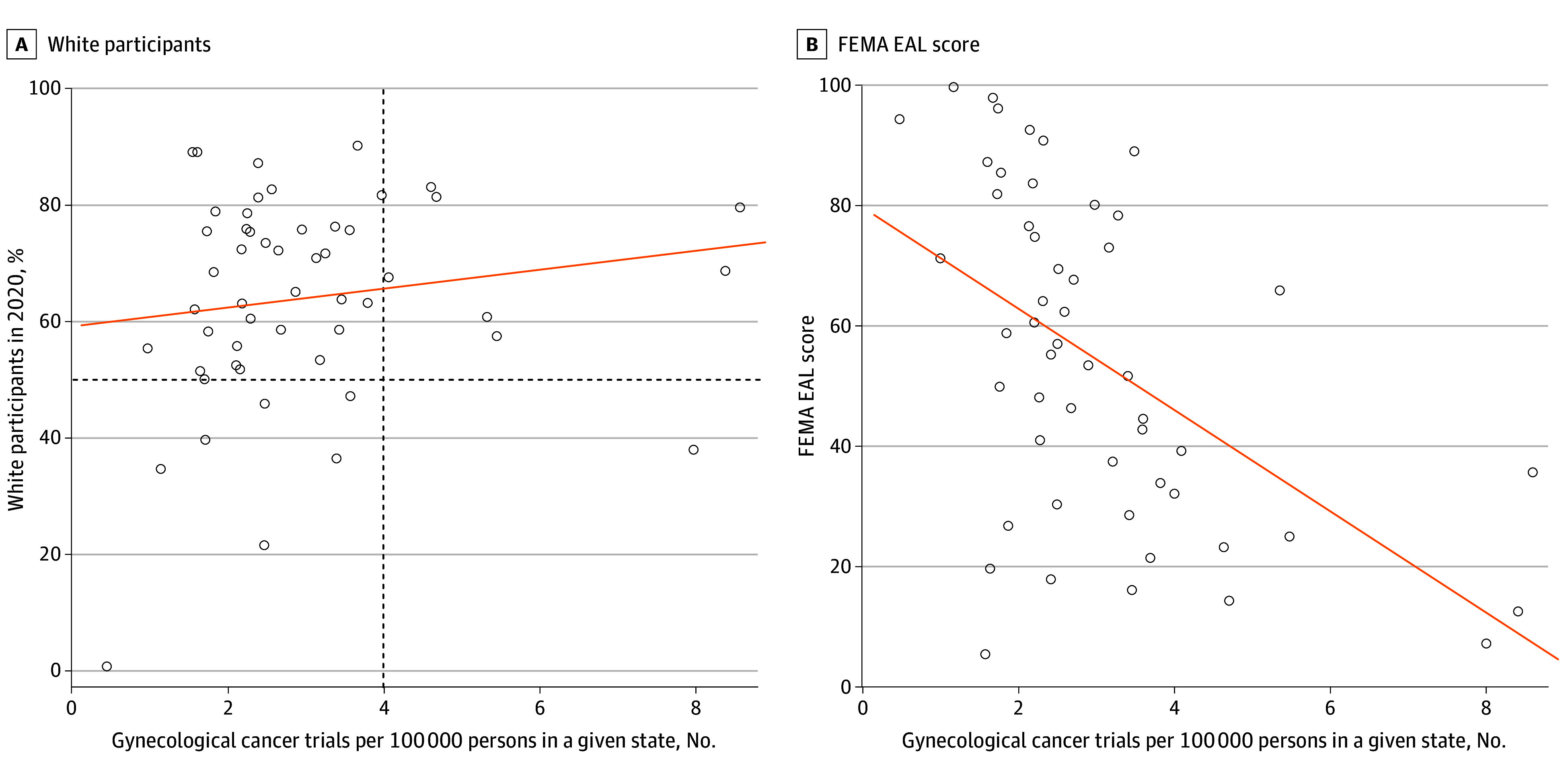
Number of Gynecological Cancer Trials Per 100 000 Persons by Percentage of Non-Hispanic White Persons and Vulnerability in the US Graphs show the association of numbers of gynecological cancer trials with the percentage of non-Hispanic White persons in the population in 2020 (A) and with the Federal Emergency Management Agency (FEMA) expected annual loss (EAL) score (B), a measure of social vulnerability across the US. Note that each point in the plots denotes a state or territory. In panel A, the dashed horizontal line represents the state-level 50% non-Hispanic White persons, and the dashed vertical line represents 4 gynecological cancer trials per 100 000 persons in a given state. Note that the lower right-hand quadrant indicating a high number of gynecological cancer trials per 100 000 persons and a low percentage of White persons in the state is empty, with the exception of one point that denotes the District of Columbia. The orange lines in A and B indicate the respective regression lines.

We then explored the association between the number of trials per 100 000 individuals living in a given state and various measures of disparities (Figure 3). We found that states and territories with more than 4 trials per 100 000 were composed of populations that were more than 50% non-Hispanic White, with the exception of District of Columbia. The correlation between the state-level percentage of non-Hispanic White persons in 2020 and the number of trials per 100 000 persons was positive, but not statistically significant (ρ = 0.20; 95% CI, −0.08 to 0.45; *P* = .16) (Figure 3A). We found that states with higher FEMA EAL had lower numbers of gynecological trials per 100 000 persons with a strong negative correlation (ρ = −0.53; 95% CI, −0.70 to −0.29; *P* < .001), indicating that states with lower EAL had higher numbers of trials gynecological cancer trials per 100 000 persons in that state (Figure 3B).

**Figure 4.**
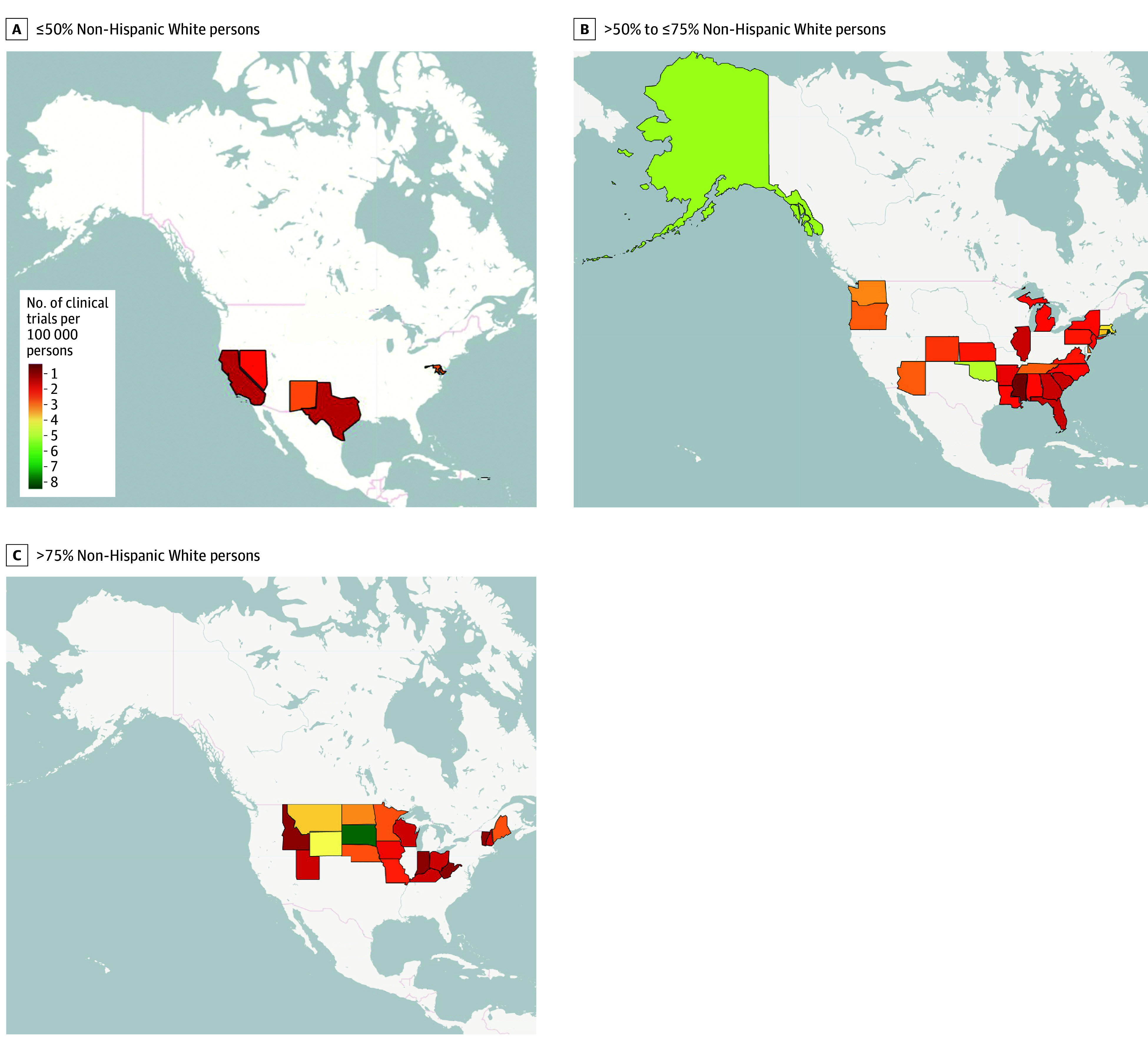
US Maps Showing Gynecological Trials Per 100 000 Persons by Percentage Non-Hispanic White Persons in the US Maps show the number of gynecological trials per 100 000 persons in states with less than or equal to 50% non-Hispanic White persons (ie, minoritized population–serving states) (A), states with greater than 50% but less than or equal to 75% non-Hispanic White persons (moderately minoritized population–serving states) (B), and in predominantly White-serving states with more than 75% non-Hispanic White persons (C). Maps were created in R using OpenStreetMap.

States with high minoritized populations and high cancer trial availability are depicted in the lower right-hand quadrant of Figure 3A. This quadrant lacks any states, indicating that there are no states with a high number of gynecological cancer trials per 100 000 persons and a high population of minoritized individuals (or low percentage of White individuals) in that same state. States with high minority populations had fewer than 4 gynecological cancer trials per 100 000 persons (Figure 4). Figure 4A reflects states with 50% or less non-Hispanic White persons, Figure 4B reflects states with more than 50% but less than or equal to 75% non-Hispanic White persons, and Figure 4C reflects predominantly White states with more than 75% non-Hispanic White persons. The dataset at the state level is available in eTable in Supplement 1 and on GitHub.^20^

## Discussion

In this cross-sectional study, we identify where in the US clinical trials for gynecological cancers are occurring and explore whether gaps exist in terms of trial access on the basis of a state’s population size, minoritized population size, and socioeconomic data (as measured using FEMA EAL). When exploring the association between where minoritized groups live in the US and trial access, we found that zero states with high minoritized populations (<50% non-Hispanic White persons) had high access to female gynecological cancer trials (as measured by 4 trials per 100 000 persons), with only the District of Columbia being the outlier.

This study’s secondary objectives include identifying states with the greatest disparities in geographic access to care as characterized by a low number of trials per 100 000 individuals living in that state. We also measured the association at the state level between the number of gynecological cancer trials per 100 000 individuals and several measures indicative of disparities, including the percentage of non-Hispanic White persons in that state and the FEMA EAL, which is used as a measure of social vulnerability in the US. We found a statistically significant association between FEMA EAL and the number of gynecological cancer trials per 100 000 (Figure 3B). For minoritized population–serving states, we found that states with higher minoritized populations (identified using the percentage of non-Hispanic White persons) had fewer trials per 100 000. Although this did not achieve statistical significance, we found that all states with 4 or more trials per 100 000 individuals had more than 50% non-Hispanic White persons in 2020 (Figure 3A), and the only exception was the District of Columbia.

There are many factors that may decrease gynecological cancer trial access in high minoritized population–serving states that we observed in this study. One factor is the lack of practitioner availability, specifically for gynecological oncology physicians in those states. A study by Ackroyd et al^21^ found that 91.2% of US counties did not have a gynecological oncologist located in their county. Although this might look stark, another study^22^ found that approximately 50% of geographic disparity issues were perceived by practitioners as resulting from or potentially posing a barrier to care. These disparities and barriers to care do not occur randomly throughout the US. There are specific regional gaps. Shalowitz et al^23^ found that counties with lower practitioner availability were also counties that tend to have higher rates of Hispanic-Latinx individuals and more low-income patients across ethnic groups.

This national problem of a lack of practitioner availability may result in patients seeking more specialized gynecological cancer care in other locations, thereby affecting referral patterns.^24,25^ Shalowitz et al^24^ found in another study that patients residing more than 50 miles away from a gynecological oncologist had increased time between referral and initiation of treatment than those living less than 50 miles away. Therefore, these factors affect whether a patient is treated by a gynecological oncologist and also referral time. It is likely that this can also affect both the time between referral and enrollment in a trial and also whether a patient is ultimately enrolled in a clinical trial.

These prior studies fit with our findings of higher minoritized populations in states with fewer trials per 100 000 persons. The state with the highest population-adjusted number of trials was South Dakota, followed by Rhode Island. Conversely, California, Mississippi, and Puerto Rico had the lowest number of trials per 100 000 persons. Two of those 3 states have high Hispanic populations, which supports prior findings.^23^

### Limitations

There are a few limitations of this study. Our study is conducted with state as the unit of analysis. There may be other lower-level factors that may contribute to trial access that were, therefore, not explored in this study. Our rationale for studying this association at the state level was because of the rarity of these gynecological cancer trials and our desire to study the association between gynecological cancers and trial availability. These data were often available at the state level and were heavily censored when we looked at more granular data. We did include 2 nonstates, the District of Columbia and Puerto Rico (a territory), which are often excluded from studies. We also included Alaska and Hawaii, which are often excluded because the majority of studies focus on the contiguous US. We wanted to include all states and Puerto Rico and District of Columbia because these have high minoritized populations and also there is increasing interest in exploring increased trial options in those regions of the US.

## Conclusions

In this cross-sectional study of female gynecological cancer trials by state, we identified gaps in gynecological cancer clinical trial availability throughout the US. We found a statistically significant association between our measure of social vulnerability (FEMA EAL) and the number of gynecological cancer trials per 100 000. We found that states with higher minoritized populations (identified using the percentage non-Hispanic White persons in each state) had fewer trials per 100 000. Although this did not achieve statistical significance, we found that all states with 4 or more trials per 100 000 individuals had more than 50% non-Hispanic White persons in 2020, and the only exception to this was the District of Columbia. Therefore, states with high minoritized populations had lower gynecological cancer trials per 100 000 persons, but there were gaps in trial availability across the entire US. Efforts are needed to address disparities identified in this study, especially the low clinical trial availability in states with high economic vulnerability and minoritized populations.

## Notes

Supplement 1. **eFigure 1.** The Breakdown of Gynecological Cancer Trials Across the USA by Cancer Site **eFigure 2.** The Number of Gynecological Cancer Trials Across the USA per Year **eFigure 3.** Relationship Between the Number of Gynecological Trials per 100,000 Female Non-Hispanic White Persons in a Given State Across the USA and the Corresponding Age-Adjusted Rates of Ovarian, Corpus Uteri, and Cervical Cancer **eTable.** The Data by State of Gynecological Cancer Trials Across the USA **eAppendix.** Details on Search Criteria

Supplement 2.
Data Sharing Statement

## References

[1] Siegel RL, Miller KD, Wagle NS, Jemal A. Cancer statistics, 2023. CA Cancer J Clin. 2023;73(1):17-48. doi:10.3322/caac.21763 36633525

[2] Collins Y, Holcomb K, Chapman-Davis E, Khabele D, Farley JH. Gynecologic cancer disparities: a report from the Health Disparities Taskforce of the Society of Gynecologic Oncology. Gynecol Oncol. 2014;133(2):353-361. doi:10.1016/j.ygyno.2013.12.039 24406291 PMC4079541

[3] The White House. Fact sheet: President Biden issues executive order and announces new actions to advance women’s health research and innovation. March 18, 2024. Accessed October 21, 2024. https://www.whitehouse.gov/briefing-room/statements-releases/2024/03/18/fact-sheet-president-biden-issues-executive-order-and-announces-new-actions-to-advance-womens-health-research-and-innovation/

[4] Rauh-Hain JA, Melamed A, Schaps D, . Racial and ethnic disparities over time in the treatment and mortality of women with gynecological malignancies. Gynecol Oncol. 2018;149(1):4-11. doi:10.1016/j.ygyno.2017.12.006 29605048

[5] US Food and Drug Administration. Enhancing the diversity of clinical trial populations—eligibility criteria, enrollment practices, and trial designs guidance for industry: FDA-2019-D-1264. November 2020. Accessed October 21, 2024. https://www.fda.gov/regulatory-information/search-fda-guidance-documents/enhancing-diversity-clinical-trial-populations-eligibility-criteria-enrollment-practices-and-trial

[6] Farley JH, Tian C, Rose GS, Brown CL, Birrer M, Maxwell GL. Race does not impact outcome for advanced ovarian cancer patients treated with cisplatin/paclitaxel: an analysis of Gynecologic Oncology Group trials. Cancer. 2009;115(18):4210-4217. doi:10.1002/cncr.24482 19536873 PMC4459778

[7] Terplan M, Temkin S, Tergas A, Lengyel E. Does equal treatment yield equal outcomes? the impact of race on survival in epithelial ovarian cancer. Gynecol Oncol. 2008;111(2):173-178. doi:10.1016/j.ygyno.2008.08.013 18823649 PMC2612941

[8] Unger JM, Moseley AB, Cheung CK, . Persistent disparity: socioeconomic deprivation and cancer outcomes in patients treated in clinical trials. J Clin Oncol. 2021;39(12):1339-1348. doi:10.1200/JCO.20.02602 33729825 PMC8078474

[9] Scully JA. Eugenics, women of color and reproductive health: the saga continues. 2004. Accessed October 21, 2024. https://papers.ssrn.com/sol3/papers.cfm?abstract_id=1649704

[10] Alsheikh B. Reproductive abuse and the sterilization of women of color: history in the making. 2023. Accessed October 21, 2024. https://scholarworks.lib.csusb.edu/cgi/viewcontent.cgi?article=1282&context=history-in-the-making

[11] Khadraoui W, Meade CE, Backes FJ, Felix AS. Racial and ethnic disparities in clinical trial enrollment among women with gynecologic cancer. JAMA Netw Open. 2023;6(12):e2346494. doi:10.1001/jamanetworkopen.2023.46494 38060227 PMC10704282

[12] Barry D, Steinberg JR, Towner M, Barber EL, Simon MA, Roque DR. Enrollment of racial and ethnic minoritized groups in gynecologic oncology clinical trials: a review of the scope of the problem, contributing factors, and strategies to improve inclusion. Clin Obstet Gynecol. 2023;66(1):22-35. doi:10.1097/GRF.0000000000000765 36657045 PMC9869456

[13] Heller C, Balls-Berry JE, Nery JD, . Strategies addressing barriers to clinical trial enrollment of underrepresented populations: a systematic review. Contemp Clin Trials. 2014;39(2):169-182. doi:10.1016/j.cct.2014.08.004 25131812 PMC6936726

[14] Segre LS, Davila RC, Carter C, Arndt S. Race/ethnicity matching boosts enrollment of black participants in clinical trials. Contemp Clin Trials. 2022;122:106936. doi:10.1016/j.cct.2022.106936 36162741

[15] Seidler EM, Keshaviah A, Brown C, Wood E, Granick L, Kimball AB. Geographic distribution of clinical trials may lead to inequities in access. Clin Invest. 2014;4(4):373-380. doi:10.4155/cli.14.21

[16] US Department of Commerce. Change in resident population of the 50 states, the District of Columbia, and Puerto Rico: 1910 to 2020. Accessed February 2024. https://web.archive.org/web/20210426202412/https://www2.census.gov/programs-surveys/decennial/2020/data/apportionment/population-change-data-table.pdf

[17] US Census Bureau. Race and ethnicity in the United States: 2010 Census and 2020 Census. August 12, 2021. Accessed February 2024. https://www.census.gov/library/visualizations/interactive/race-and-ethnicity-in-the-united-state-2010-and-2020-census.html

[18] Wikipedia. List of U.S. states by non-Hispanic white population. Accessed February 2024. https://en.wikipedia.org/wiki/List_of_U.S._states_by_non-Hispanic_white_population#cite_note-8

[19] Federal Emergency Management Agency. Expected annual loss. Accessed February 2024. https://hazards.fema.gov/nri/expected-annual-loss

[20] Boland Lab. GitHub. Accessed October 25, 2024. https://github.com/bolandlab/Boland_GynOncTrials_JAMAOpen

[21] Ackroyd SA, Shih YT, Kim B, Lee NK, Halpern MT. A look at the gynecologic oncologist workforce: are we meeting patient demand? Gynecol Oncol. 2021;163(2):229-236. doi:10.1016/j.ygyno.2021.08.013 34456058 PMC8585725

[22] Ricci S, Tergas AI, Long Roche K, . Geographic disparities in the distribution of the U.S. gynecologic oncology workforce: a Society of Gynecologic Oncology study. Gynecol Oncol Rep. 2017;22:100-104. doi:10.1016/j.gore.2017.11.006 29201989 PMC5699889

[23] Shalowitz DI, Vinograd AM, Giuntoli RL II. Geographic access to gynecologic cancer care in the United States. Gynecol Oncol. 2015;138(1):115-120. doi:10.1016/j.ygyno.2015.04.025 25922191

[24] Shalowitz D, Madeka I, Evans J, . Referral patterns for gynecologic oncology consultation. Gynecol Oncol. 2021;162:S260-S61. doi:10.1016/S0090-8258(21)01144-6

[25] Madeka I, Burnett BA, Shalowitz DI. Referral patterns for gynecologic oncology consultation. Obstet Gynecol. 2019;133:49S-51S. doi:10.1097/01.AOG.0000558985.80976.2a

