# Supplementary material for: Geographic Disparities in Gynecologic Oncology Clinical Trial Availability in the US
Source: JAMA Netw Open. 2024 Nov 26;7(11):e2447635. doi: 10.1001/jamanetworkopen.2024.47635 (PMC11600227; doi:10.1001/jamanetworkopen.2024.47635)
Supplement: Supplement 1. — eFigure 1. The Breakdown of Gynecological Cancer Trials Across the USA by Cancer Site eFigure 2. The Number of Gynecological Cancer Trials Across the USA per Year eFigure 3. Relationship Between the Number of Gynecological Trials per 100,000 Female Non-Hispanic White Persons in a Given State Across the USA and the Corresponding Age-Adjusted Rates of Ovarian, Corpus Uteri, and Cervical Cancer eTable. The Data by State of Gynecological Cancer Trials Across the USA eAppendix. Details on Search Criteria [file jamanetwopen-e2447635-s001.pdf]

Supplemental Online Content

Boland MR, Tubridy E, Solorzano SS, Simpkins F, Smith AJB, Ko EM. Geographic disparities in gynecologic oncology clinical trial availability in the US. *JAMA Netw Open*. 2024;7(11):e2447635. doi:10.1001/jamanetworkopen.2024.47635

eFigure 1. The Breakdown of Gynecological Cancer Trials Across the USA by Cancer Site
eFigure 2. The Number of Gynecological Cancer Trials Across the USA per Year
eFigure 3. Relationship Between the Number of Gynecological Trials per 100,000 Female Non-Hispanic White Persons in a Given State Across the USA and the Corresponding Age-Adjusted Rates of Ovarian, Corpus Uteri, and Cervical Cancer
eTable. The Data by State of Gynecological Cancer Trials Across the USA
eAppendix. Details on Search Criteria

This supplemental material has been provided by the authors to give readers additional information about their work.

© 2024 Boland MR et al. *JAMA Netw Open.*

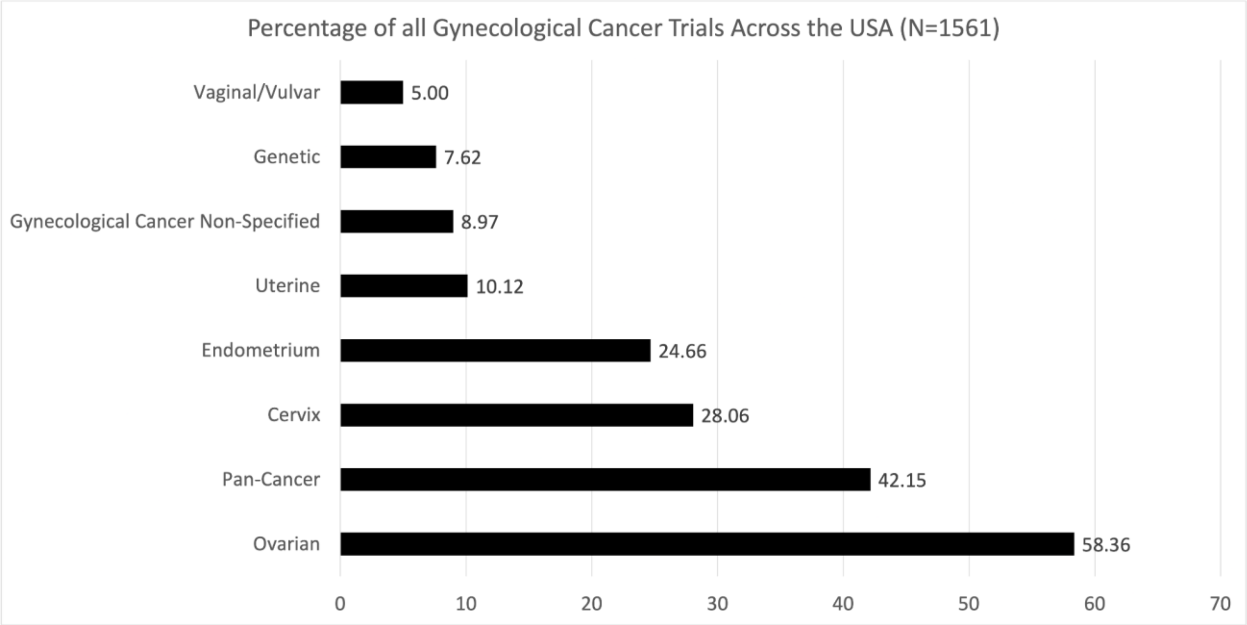

eFigure 1. The Breakdown of Gynecological Cancer Trials Across the USA by Cancer Site
Caption: The percentage is provided for each gynecological cancer site out of the total of 1,561 trials. Note that Ovarian cancer has the highest percentage of trials with 58.36%. Note that Uterine trials are non-endometrial uterine trials.

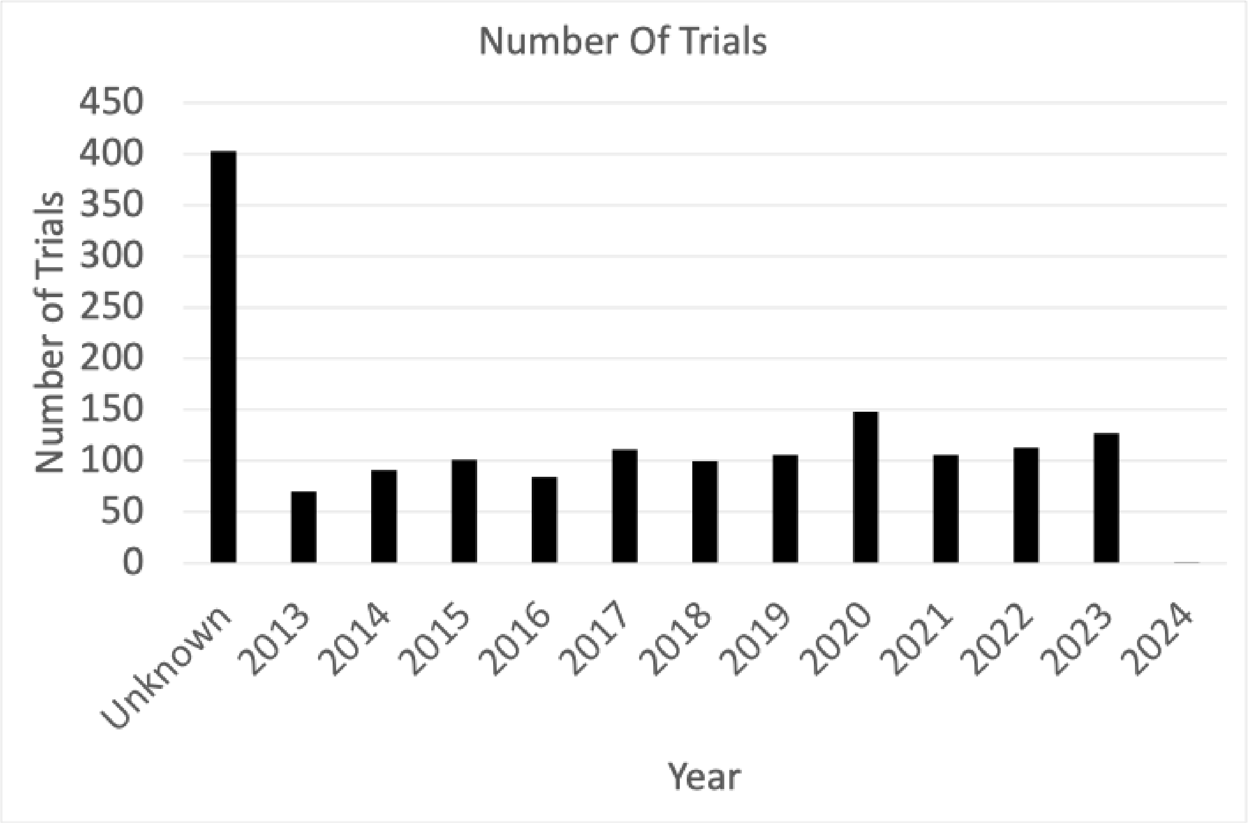

eFigure 2. The Number of Gynecological Cancer Trials Across the USA per Year. Some Trials had an Unknown Year because it was not easily extractable from the Data and those are listed as 'Unknown' above.

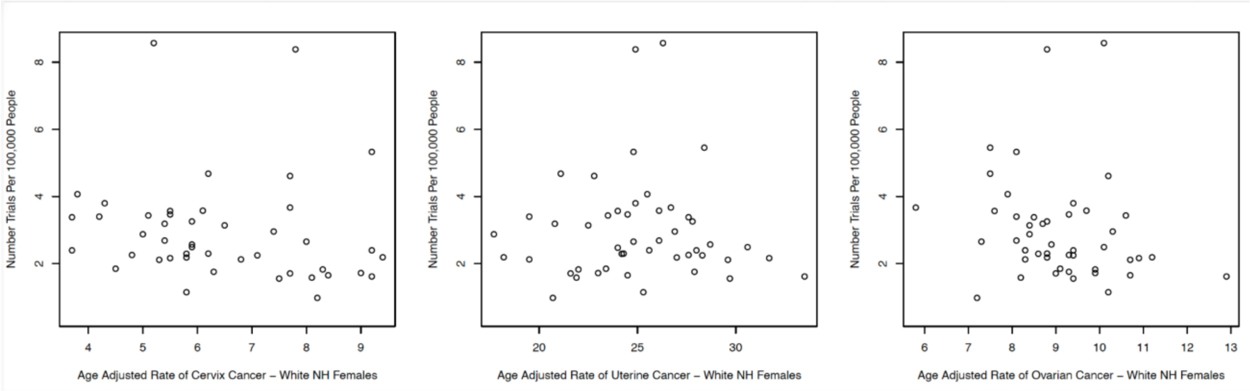

eFigure 3. Relationship Between the Number of Gynecological Trials per 100,000 Female Non-Hispanic White Persons in a Given State Across the USA and the Corresponding Age-Adjusted Rates of Ovarian, Corpus Uteri, and Cervical Cancer. Note that each point in the plot above denotes a state.

© 2024 Boland MR et al. *JAMA Netw Open.*

eTable. The Data by State of Gynecological Cancer Trials Across the USA

GEO ID	State	Num. Trials per State	Census Pop. Apr 1, 2020	Num. Trials per State per 100,000 persons	Cervix*	Uterine*	Ovarian*	region	% White	FEMA EAL	Num Obs. Trials per State	Num Inter. Trials per State
1	Alabama	110	5024279	2.19	9.4	18.2	11.2	South	63.1	75	7	102
2	Alaska	40	733391	5.45	NA	28.4	7.5	West	57.5	25	2	38
4	Arizona	228	7151502	3.19	5.4	20.8	8.7	West	53.4	37.5	11	217
5	Arkansas	55	3011524	1.83	8.3	22	9.9	South	68.5	58.93	6	49
6	California	454	39538223	1.15	5.8	25.3	10.2	West	34.7	100	32	420
8	Colorado	166	5773714	2.88	5	17.7	8.4	West	65.1	53.57	7	159
9	Connecticut	137	3605944	3.80	4.3	24.9	9.4	Northeast	63.2	33.93	8	129
10	Delaware	34	989948	3.43	5.1	23.5	10.6	South	58.6	16.07	3	31
11	District of Columbia	55	689545	7.98	NA	25.9	NA	South	38	7.14	3	52
12	Florida	356	21538187	1.65	8.4	24.5	10.7	South	51.5	98.21	30	325
13	Georgia	183	10711908	1.71	7.7	21.6	9	South	50.1	82.14	14	169
15	Hawaii	36	1455271	2.47	NA	24	NA	West	21.6	30.36	4	32
16	Idaho	34	1839106	1.85	4.5	23.4	9.1	West	78.9	26.79	4	30
17	Illinois	225	12812508	1.76	6.3	27.9	9.3	Midwest	58.3	85.71	18	206
18	Indiana	118	6785528	1.74	NA	NA	NA	Midwest	75.5	50	9	109
19	Iowa	82	3190369	2.57	5.9	28.7	8.9	Midwest	82.7	62.5	9	73
20	Kansas	78	2937880	2.65	8	24.8	7.3	Midwest	72.2	46.43	5	73
21	Kentucky	108	4505836	2.40	9.2	25.6	8.3	South	81.3	55.36	7	101
22	Louisiana	99	4657757	2.13	6.8	19.5	8.3	South	55.8	92.86	8	91
23	Maine	50	1362359	3.67	7.7	26.7	5.8	Northeast	90.2	21.43	5	44
24	Maryland	221	6177224	3.58	6.1	26.1	9.7	South	47.2	44.64	17	203
25	Massachusetts	286	7029917	4.07	3.8	25.5	7.9	Northeast	67.6	39.29	13	273

© 2024 Boland MR et al. *JAMA Netw Open.*

26	Michigan	220	10077331	2.18	5.8	27	8.8	Midwest	72.4	60.71	10	210
27	Minnesota	193	5706494	3.38	3.7	27.6	8.5	Midwest	76.3	51.79	21	171
28	Mississippi	29	2961279	0.98	8.2	20.7	7.2	South	55.4	71.43	2	27
29	Missouri	182	6154913	2.96	7.4	26.9	10.3	Midwest	75.8	80.36	12	169
30	Montana	50	1084225	4.61	7.7	22.8	10.2	West	83.1	23.21	3	47
31	Nebraska	70	1961504	3.57	5.5	24	7.6	Midwest	75.7	42.86	7	63
32	Nevada	77	3104614	2.48	NA	NA	NA	West	45.9	57.14	3	74
33	New Hampshire	33	1377529	2.40	3.7	28	9.4	Northeast	87.2	17.86	3	30
34	New Jersey	201	9288994	2.16	5.5	31.7	10.9	Northeast	51.8	83.93	17	184
35	New Mexico	72	2117522	3.40	4.2	19.5	8.1	West	36.5	28.57	4	67
36	New York	427	20201249	2.11	5.3	29.6	10.7	Northeast	52.5	76.79	33	394
37	North Carolina	240	10439388	2.30	6.2	24.3	8.8	South	60.5	91.07	28	211
38	North Dakota	31	779094	3.98	NA	NA	NA	Midwest	81.7	32.14	4	27
39	Ohio	265	11799448	2.25	7.1	28.3	9.4	Midwest	75.9	48.21	17	247
40	Oklahoma	211	3959353	5.33	9.2	24.8	8.1	South	60.8	66.07	7	204
41	Oregon	138	4237256	3.26	5.9	27.8	8.8	West	71.7	78.57	7	130
42	Pennsylvania	324	13002700	2.49	5.9	30.6	10.1	Northeast	73.5	69.64	22	302
72	Puerto Rico	15	3285874	0.46	NA	NA	NA	NA	0.8	94.64	2	13
44	Rhode Island	92	1097379	8.38	7.8	24.9	8.8	Northeast	68.7	12.5	5	87
45	South Carolina	81	5118425	1.58	8.1	21.9	8.2	South	62.1	87.5	9	72
46	South Dakota	76	886667	8.57	5.2	26.3	10.1	Midwest	79.6	35.71	5	71
47	Tennessee	217	6910840	3.14	6.5	22.5	8.4	South	70.9	73.21	8	209
48	Texas	501	29145505	1.72	9	23	9.9	South	39.7	96.43	32	467

© 2024 Boland MR et al. *JAMA Netw Open.*

49	Utah	75	3271616	2.29	5.8	24.2	8.6	West	75.4	64.29	3	72
50	Vermont	10	643077	1.56	7.5	29.7	9.4	Northeast	89.1	5.36	2	8
51	Virginia	232	8631393	2.69	5.4	26.1	8.1	South	58.6	67.86	13	219
53	Washington	267	7705281	3.47	5.5	24.5	9.3	West	63.8	89.29	17	250
54	West Virginia	29	1793716	1.62	9.2	33.5	12.9	South	89.1	19.64	2	27
55	Wisconsin	133	5893718	2.26	4.8	27.6	9.3	Midwest	78.6	41.07	10	122
56	Wyoming	27	576851	4.68	6.2	21.1	7.5	West	81.4	14.29	4	23

***** Age-Adjusted Rates from the CDC for each of these cancers respectively among non-Hispanic White females

Abbreviations:
Pop.: Population
Inter.: Interventional
Obs.: Observational
Num.: Number
NA.: Not Available

© 2024 Boland MR et al. *JAMA Netw Open.*

eAppendix. Details on Search Criteria

January 10, 2024: Entire Country Search (Dr. Mary Regina Boland)

- **Search 1:**
  - **§ Condition or disease: Ovarian cancer**
  - **§ Location: United States**
  - **§ All ages, All sexes, All phases**
  - **§ First posted from: 01/01/2013**
  - **§ CSV Including: NCT Number, Study Title, URL, Study status, Conditions, Interventions, Primary outcome, Phases, Enrollment, Study Type, Date First Posted, Locations**
- **Search 2:**
  - **§ Condition or disease: Uterine cancer**
  - **§ Location: United States**
  - **§ All ages, All sexes, All phases**
  - **§ First posted from: 01/01/2013**
  - **§ CSV Including: NCT Number, Study Title, URL, Study status, Conditions, Interventions, Primary outcome, Phases, Enrollment, Study Type, Date First Posted, Locations**
- **Search 3:**
  - **§ Condition or disease: Cervical cancer**
  - **§ Location: United States**
  - **§ All ages, All sexes, All phases**
  - **§ First posted from: 01/01/2013**
  - **§ CSV Including: NCT Number, Study Title, URL, Study status, Conditions, Interventions, Primary outcome, Phases, Enrollment, Study Type, Date First Posted, Locations**
- **Search 4:**
  - **§ Condition or disease: Endometrial cancer**
  - **§ Location: United States**
  - **§ All ages, All sexes, All phases**
  - **§ First posted from: 01/01/2013**
  - **§ CSV Including: NCT Number, Study Title, URL, Study status, Conditions, Interventions, Primary outcome, Phases, Enrollment, Study Type, Date First Posted, Locations**
- **Search 5:**
  - **§ Condition or disease: Gynecologic cancer**
  - **§ Location: United States**
  - **§ All ages, All sexes, All phases**
  - **§ First posted from: 01/01/2013**
  - **§ CSV Including: NCT Number, Study Title, URL, Study status, Conditions, Interventions, Primary outcome, Phases, Enrollment, Study Type, Date First Posted, Locations**

© 2024 Boland MR et al. *JAMA Netw Open.*

- **Search 6:**
  - **§ Condition or disease: uterine stromal cancer**
  - **§ Location: United States**
  - **§ All ages, All sexes, All phases**
  - **§ First posted from: 01/01/2013**
  - **§ CSV Including: NCT Number, Study Title, URL, Study status, Conditions, Interventions, Primary outcome, Phases, Enrollment, Study Type, Date First Posted, Locations**
- **Search 7:**
  - **§ Condition or disease: ovarian germ cell cancer**
  - **§ Location: United States**
  - **§ All ages, All sexes, All phases**
  - **§ First posted from: 01/01/2013**
  - **§ CSV Including: NCT Number, Study Title, URL, Study status, Conditions, Interventions, Primary outcome, Phases, Enrollment, Study Type, Date First Posted, Locations**
  - **§**
- **Search 8:**
  - **§ Condition or disease: uterine sarcoma**
  - **§ Location: United States**
  - **§ All ages, All sexes, All phases**
  - **§ First posted from: 01/01/2013**
  - **§ CSV Including: NCT Number, Study Title, URL, Study status, Conditions, Interventions, Primary outcome, Phases, Enrollment, Study Type, Date First Posted, Locations**
  - **§**
- **Search 9:**
  - **§ Condition or disease: fallopian tube cancer**
  - **§ Location: United States**
  - **§ All ages, All sexes, All phases**
  - **§ First posted from: 01/01/2013**
  - **§ CSV Including: NCT Number, Study Title, URL, Study status, Conditions, Interventions, Primary outcome, Phases, Enrollment, Study Type, Date First Posted, Locations**
- **Search 10:**
  - **§ Condition or disease: primary peritoneal cancer**
  - **§ Location: United States**
  - **§ All ages, All sexes, All phases**
  - **§ First posted from: 01/01/2013**
  - **§ CSV Including: NCT Number, Study Title, URL, Study status, Conditions, Interventions, Primary outcome, Phases, Enrollment, Study Type, Date First Posted, Locations**
- **Search 11:**
  - **§ Condition or disease: vulvar cancer**

© 2024 Boland MR et al. *JAMA Netw Open.*

§ Location: United States
§ All ages, All sexes, All phases
§ First posted from: 01/01/2013
§ CSV Including: NCT Number, Study Title, URL, Study status, Conditions, Interventions, Primary outcome, Phases, Enrollment, Study Type, Date First Posted, Locations

- **Search 12:**
  - **§ Condition or disease: Vaginal cancer**
  - **§ Location: United States**
  - **§ All ages, All sexes, All phases**
  - **§ First posted from: 01/01/2013**
  - **§ CSV Including: NCT Number, Study Title, URL, Study status, Conditions, Interventions, Primary outcome, Phases, Enrollment, Study Type, Date First Posted, Locations**

© 2024 Boland MR et al. *JAMA Netw Open.*
