# Supplementary material for: Geographic Disparities in Gynecologic Oncology Clinical Trial Availability in the US
Source: JAMA Netw Open. 2024 Nov 26;7(11):e2447635. doi: 10.1001/jamanetworkopen.2024.47635 (PMC11600227; doi:10.1001/jamanetworkopen.2024.47635)
Supplement: Supplement 2. — Data Sharing Statement [file jamanetwopen-e2447635-s002.pdf]

Data Sharing Statement

Boland. Geospatial Disparities in Gynecologic Oncology Clinical Trial Availability in the US. *JAMA Netw Open*. Published November 26, 2024. doi:10.1001/jamanetworkopen.2024.47635

Data
Data available: No

Additional Information
Explanation for why data not available: We will make data available that is possible via Github due to privacy concerns. Most of our data uses ClinicalTrials.gov so those data should be able to be made publicly available.
